# Supplementary material for: Reciprocal modulation of ammonia and melanin production has implications for cryptococcal virulence
Source: Nat Commun. 2023 Feb 15;14:849. doi: 10.1038/s41467-023-36552-7 (PMC9932161; doi:10.1038/s41467-023-36552-7)
Supplement: Supplementary file 2 — Reporting Summary [file 41467_2023_36552_MOESM2_ESM.pdf]

nature portfolio

Corresponding author(s): Arturo Casadevall

Last updated by author(s): Jan 23, 2023

Reporting Summary

Nature Portfolio wishes to improve the reproducibility of the work that we publish. This form provides structure for consistency and transparency in reporting. For further information on Nature Portfolio policies, see our Editorial Policies and the Editorial Policy Checklist.

Statistics

For all statistical analyses, confirm that the following items are present in the figure legend, table legend, main text, or Methods section.

n/a	Confirmed	
☐	☒	The exact sample size (*n*) for each experimental group/condition, given as a discrete number and unit of measurement
☐	☒	A statement on whether measurements were taken from distinct samples or whether the same sample was measured
☐	☒	repeatedly The statistical test(s) used AND whether they are one- or two-sided<br>*Only common tests should be described solely by name; describe more complex techniques in the Methods section.*
☒	☐	A description of all covariates tested
☐	☒	A description of any assumptions or corrections, such as tests of normality and adjustment for multiple comparisons
☐	☒	A full description of the statistical parameters including central tendency (e.g. means) or other basic estimates (e.g. regression coefficient) AND variation (e.g. standard deviation) or associated estimates of uncertainty (e.g. confidence intervals)
☐	☒	For null hypothesis testing, the test statistic (e.g. *F*, *t*, *r*) with confidence intervals, effect sizes, degrees of freedom and *P* value noted<br>*Give P values as exact values whenever suitable.*
☒	☐	For Bayesian analysis, information on the choice of priors and Markov chain Monte Carlo settings
☒	☐	For hierarchical and complex designs, identification of the appropriate level for tests and full reporting of
☒	☐	outcomes Estimates of effect sizes (e.g. Cohen's *d*, Pearson's *r*), indicating how they were calculated

Our web collection on statistics for biologists contains articles on many of the points above.

Software and code

Policy information about availability of computer code

Data collection	Softmax Pro 7.1 software was used to measure absorbance and fluorescence with a Spectramax iD5 microplate reader and QCapture-Pro 6.0 software was used to obtain microscopy images.
Data analysis	Adobe Photoshop 2021 was used to crop images and to convert color photographs to grayscale images prior to quantification of pigmentation. Image Studio Lite 5.2 software was used to quantify pixel intensity for pigmentation analysis. Numerical data were graphed and analyzed for statistical significance using GraphPad Prism 9 software.

For manuscripts utilizing custom algorithms or software that are central to the research but not yet described in published literature, software must be made available to editors and reviewers. We strongly encourage code deposition in a community repository (e.g. GitHub). See the Nature Portfolio guidelines for submitting code & software for further information.

Data

Policy information about availability of data

All manuscripts must include a data availability statement. This statement should provide the following information, where applicable:

- Accession codes, unique identifiers, or web links for publicly available datasets
- A description of any restrictions on data availability
- For clinical datasets or third party data, please ensure that the statement adheres to our policy

The authors declare that the data supporting the findings of this study are available within the article. Source data are provided with this paper.

Human research participants

Policy information about [studies involving human research participants and Sex and Gender in Research](#).

Reporting on sex and gender	No human research participants were involved in this study.
Population characteristics	N/A
Recruitment	N/A
Ethics oversight	N/A

Note that full information on the approval of the study protocol must also be provided in the manuscript.

Field-specific reporting

Please select the one below that is the best fit for your research. If you are not sure, read the appropriate sections before making your selection.

☒ Life sciences ☐ Behavioural & social sciences ☐ Ecological, evolutionary & environmental sciences

For a reference copy of the document with all sections, see nature.com/documents/nr-reporting-summary-flat.pdf

Life sciences study design

All studies must disclose on these points even when the disclosure is negative.

Sample size	Sample sizes of at least 3 or 4 biological replicates were chosen based on previous experience and published protocols.
Data exclusions	In the experiment described in Fig. 4, two of the ten mice infected with ure1Δ cells failed to establish an infection and were excluded from the analysis.
Replication	Independent biological replicates were performed as described in the Methods and Figure legends. All replication attempts were successful and gave similar results.
Randomization	For animal studies, mice were chosen randomly for infection with either WT or mutant C. neoformans.
Blinding	Blinding was not relevant to this study as experiments generated quantitative measurements and did not require qualitative analyses.

Reporting for specific materials, systems and methods

We require information from authors about some types of materials, experimental systems and methods used in many studies. Here, indicate whether each material, system or method listed is relevant to your study. If you are not sure if a list item applies to your research, read the appropriate section before selecting a response.

Materials & experimental systems

n/a	Involved in the study	
☒	☐	Antibodies
☒	☐	Eukaryotic cell lines
☒	☐	Palaeontology and archaeology
☐	☒	Animals and other organisms
☒	☐	Clinical data
☒	☐	Dual use research of concern

Methods

n/a	Involved in the study	
☒	☐	ChIP-seq
☒	☐	Flow cytometry
☒	☐	MRI-based neuroimaging

Animals and other research organisms

Policy information about [studies involving animals](#); [ARRIVE guidelines](#) recommended for reporting animal research, and [Sex and Gender in Research](#)

Laboratory animals	Female C57BL/6J mice, aged 5-7 weeks

Wild animals	No wild animals were used in this study.
Reporting on sex	The sex of model animals was chosen for conformity to established protocols for C. neoformans infections and is not considered to influence the outcome of experiments.
Field-collected samples	No field-collected samples were used in this study.
Ethics oversight	Animal work was performed under protocol number MO21H124, as approved by Johns Hopkins University Animal Care and Use Committee.

Note that full information on the approval of the study protocol must also be provided in the manuscript.

This checklist template is licensed under a Creative Commons Attribution 4.0 International License, which permits use, sharing, adaptation, distribution and reproduction in any medium or format, as long as you give appropriate credit to the original author(s) and the source, provide a link to the Creative Commons license, and indicate if changes were made. The images or other third party material in this article are included in the article's Creative Commons license, unless indicated otherwise in a credit line to the material. If material is not included in the article's Creative Commons license and your intended use is not permitted by statutory regulation or exceeds the permitted use, you will need to obtain permission directly from the copyright holder. To view a copy of this license, visit http://creativecommons.org/licenses/by/4.0/
